# Prevalences and Indications of Psychotropic Drug Prescriptions in Nursing Home Residents with Korsakoff Syndrome

**DOI:** 10.3390/jcm12093133

**Published:** 2023-04-26

**Authors:** Ineke J. Gerridzen, Els Doejaaren, Ruth B. Veenhuizen, Cees M. P. M. Hertogh, Karlijn J. Joling

**Affiliations:** 1 Atlant, Korsakoff Centre of Expertise, Kuiltjesweg 1, 7361 TC Beekbergen, The Netherlands; 2 Amsterdam UMC, Location Vrije Universiteit Amsterdam, Department of Medicine for Older People, De Boelelaan 1109, 1081 HV Amsterdam, The Netherlands; 3 Amsterdam Public Health, Aging & Later Life, De Boelelaan 1109, 1081 HV Amsterdam, The Netherlands

**Keywords:** Korsakoff syndrome, nursing home, psychotropic drugs, behavioral symptoms

## Abstract

Psychotropic drugs (PD) are often prescribed to nursing home residents with Korsakoff syndrome (KS). It is unknown whether these drugs are prescribed correctly or whether they are prescribed off-label, for example, to treat behavioral symptoms. To get more insight into PD prescriptions, a descriptive study was performed. The type, category and indications of PD prescriptions of 285 participants were analyzed using medication charts and questionnaires. Behavioral symptoms were investigated with the Neuropsychiatric Inventory-Questionnaire. The results showed that atypical antipsychotics (57.1%) were prescribed more frequently than typical antipsychotics (49.3%). Of the antidepressants, selective serotonin/norepinephrine reuptake inhibitors (63.1%) were most frequently prescribed, followed by tricyclic antidepressants (23.4%). Of the benzodiazepines, anxiolytics (85.7%) were more prescribed than hypnotics (24.5%). Besides psychiatric disorders, PD were also prescribed to treat behavioral symptoms varying from 29.9% (antipsycho-tics) to 26.3% (benzodiazepines) and 9.3% (antidepressants). Furthermore, prescriptions were high if behavioral symptoms were present. To conclude, PD are often prescribed to residents with KS for an unapproved indication, namely behavioral symptoms. Additional research is needed to obtain further insight into the current prescribing culture and the effectiveness of PD. The insights thus obtained may, ultimately, contribute to the appropriate prescription of PD for people with KS.

## 1. Introduction

The neuropsychiatric disorder Korsakoff syndrome (KS) is caused by thiamine deficiency, which, in particular, occurs among alcoholics. The key feature of KS is severe memory impairment, which manifests as anterograde and retrograde amnesia. Current and recent memory is affected more than remote memory; however, the impairment could involve memories from up to 30 years earlier. The memory deficits are primarily related to declarative memory. In particular, episodic memory is severely affected. Executive functioning is also commonly affected. The cognitive deficits are often associated with confabulations [1,2]. In the Netherlands, there is a long tradition of nursing home care for people with severe forms of KS, and many of them reside in specialized nursing homes (NH). These residents are a specific subgroup that differs from the general NH population: they are often relatively young, single or divorced men with much somatic and psychiatric comorbidity [3,4]. Behavioral symptoms are also highly prevalent and often occur concomitantly. In particular, irritability, agitation and disinhibition are common [4]. In addition, impaired awareness in performing everyday tasks is another typical feature and may lead to a rejection of care [5,6,7,8]. These features challenge the skills and capacity of professional caregivers in supporting these residents. In fact, in daily practice, caregivers report that providing good care to these residents is often quite a battle [9]. This, in turn, may lead to the inappropriate prescription of psychotropic drugs (PD).

Although treating behavioral symptoms of NH residents with dementia through the prescription of PD has now proven controversial because of considerable side effects and unproven effectiveness [10,11,12], more than two-thirds of NH residents with KS use these drugs. In particular, antipsychotics are prescribed very often (47%), followed by antidepressants (38%) and benzodiazepines (36%) [3,4]. In addition, a great variation in the patterns of psychotropic drug prescriptions (PDP) between the different NH was found [3]. These findings cannot be fully explained by the prevalence of comorbid psychiatric disorders. However, to date, it is unknown for which indications exactly are PD prescribed to these residents. As in residents with dementia, it is therefore suggested that PD are inappropriately prescribed, not only for the treatment of psychiatric disorders but probably also for the treatment of behavioral symptoms in KS residents [3,4].

In the Netherlands, trained elderly care physicians are involved in the care of NH residents with KS and all of them are employed by the NH. As a result, these physicians have a lot of clinical experience in the care and treatment of these residents. However, scientific research is scarce and specific guidelines for the treatment of behavioral symptoms and appropriate prescription of psychotropic drugs (PDP) for these residents are lacking. Moreover, the effectiveness and side effects of PDP in people with KS are unknown. Therefore, the aim of this study was to gain insight into the categories, types and indications of PD that are prescribed to NH residents with KS. In addition, the present study aimed also to explore the relationship between PDP and behavioral symptoms. The insights thus obtained may be a first step in disentangling the current prescribing culture of PD for people with KS.

## 2. Materials and Methods

### 2.1. Study Design, Setting and Participants

An in-depth study was performed using data from the study on the prevalence and severity of neuropsychiatric symptoms in NH residents with KS, which was already published [4]. In this cross-sectional, descriptive study that was performed from September 2014 to February 2016, more detailed data were obtained about the prevalence of PD prescriptions and their indications.

Participants were selected according to the following inclusion criteria: (1) a primary diagnosis of KS, Wernicke encephalopathy, Wernicke-Korsakoff syndrome, alcohol-induced persisting amnestic disorder, alcohol-induced persisting dementia, alcohol-related dementia, alcoholic dementia or alcohol-related persistent cognitive impairment as reported by the physician in the medical record. Due to the lack of clear definition criteria, several terms are used interchangeably to indicate KS. As this hampers a clear description of patients or study groups, in the remainder of this article, we refer to these diagnoses with the umbrella term KS; (2) being admitted for at least 3 months and (3) availability of a legal representative to give informed consent.

Eligible participants were included in the study after written informed consent of both the resident himself and the legal representative was obtained. If the resident did not have the capacity to make an informed decision, only written informed consent of the legal representative was asked. The institutional review board of the VU University Me-dical Center Amsterdam approved the research protocol and considered it not to be subject to the Dutch Medical Research Involving Human Subjects Act (WC 2014-010 on 30 January 2014). Data were stored at Amsterdam UMC and were only accessible through a secured network environment by the research team.

### 2.2. Measurements

Baseline data on psychotropic drug prescriptions were assessed using two methods: (1) Review of medication charts retrieved from the medication administration record. This review provided details about the category and type of PD. ‘As needed’ PDP were not included. And (2) Questionnaire, which provided details about the category and indication of PD. For each category, the treating physician was asked if there was a PDP (‘yes’ or ‘no’). If ‘yes’, the indication of PDP was asked. These indications were defined by the elderly care physicians of the research group (C.H., R.V. and I.G.) and were based on DSM-IV-TR diagnoses [13] and clinical practice. It was possible to fill in more than one indication. Indications that could be chosen for antipsychotics were schizophrenia or other psychotic disorder, behavioral symptoms or other. Indications that could be chosen for antidepressants were mood disorder, anxiety disorder, obsessive compulsive disorder, behavioral symptoms or other. Indications that could be chosen for benzodiazepines were anxiety, sleep disorder, behavioral symptoms or other.

Psychotropic drugs were classified according to the Anatomical Therapeutic Chemical (ATC) Classification System of the WHO [14]. Next, they were grouped into three major categories and subdivided into types: antipsychotics (typical or atypical antipsychotic), antidepressants (selective serotonin/norepinephrine reuptake inhibitor (SSRI/SNRI), tricyclic antidepressant (TCA) or other antidepressants) and benzodiazepines (anxiolytic or hypnotic).

Sociodemographic and clinical characteristics on age, gender, education, marital status, length of stay and somatic and psychiatric comorbidity were retrieved from residents’ medical records.

Cognitive functioning was assessed with the Cognitive Performance Scale (CPS), which measures a resident’s everyday cognitive performance in four domains: short term memory, daily decision-making, the ability to make oneself understood and the ability to feed independently. The total CPS score is calculated by using a hierarchical scoring system and ranges from 0 (intact) to 6 (very severe impairment). The CPS scores are categorized into no or mild impairment (CPS 0–1), moderate impairment (CPS 2–4) and severe impairment (CPS 5–6). [15].

Behavioral symptoms were assessed with the Neuropsychiatric Inventory-Questionnaire (NPI-Q). The NPI-Q provides information on 12 behavioral and psychological symptom domains. After the screening question to determine whether the symptom had been “absent” (score = 0) or “present” (score = 1) in the last month, severity was scored on a 3-point scale ranging from 1 (mild) to 3 (severe). The NPI-Q total severity score is the sum of the severity scores for each symptom ranging from 0 to 36, with higher scores indicating more severe symptoms [16].

### 2.3. Data Collection

To obtain data on sociodemographic and clinical characteristics, the elderly care physician was asked to complete an online survey, which also included the questionnaire about PD category and indication. The second author (E.D., elderly care physician in training) investigated PD category and type through a review of residents’ medication charts. Research interviewers and a research assistant, all trained by the first author (I.G.), admi-nistered the CPS and NPI-Q to the primary responsible nurse or nurse assistant of the residents.

### 2.4. Statistical Analysis

Descriptive statistics (numbers, percentages, means, standard deviations and ranges) were used to calculate residents’ characteristics and the prevalence of PDP. To check the actual use of PD and to explore if there were discrepancies, PDP, as filled in by the physician in the questionnaire, were compared to the resident’s medication charts. By using cross tabulation, the relationship between behavioral symptoms and PDP was explored. All data were analyzed using SPSS Statistics 28.

## 3. Results

The study sample consisted of 285 residents. A total of 283 medication charts were retrieved from residents’ medication administration records. The elderly care physicians completed 282 questionnaires on categories of PD. Because one physician resigned during the study, data on indications of PDP were missing from 35 residents, resulting in 247 questionnaires on indications.

### 3.1. Sociodemographic and Clinical Characteristics

The mean age was 63 years. Most residents were male (78%) and single (86%). The mean length of stay was 6.5 years. Cardiovascular diseases, neurological diseases, chronic obstructive pulmonary disease (COPD) and mood disorders were the most common comorbidities. Cognitive functioning was moderately impaired (Table 1).

### 3.2. Prevalence of Psychotropic Drug Prescriptions

Table 2 shows the prevalences of the categories, types and combinations of PDP according to the medication charts versus the questionnaires. No major differences were seen between the medication charts and the questionnaires. We present the percentages below for the medication charts.

**Figure 1:**
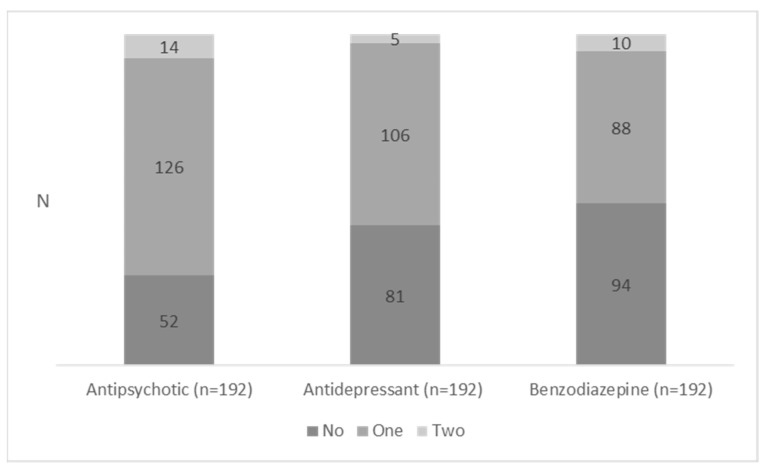
Number of prescribed psychotropic drugs according to medication chart (N = 283).

PD were prescribed to two-thirds of residents (n = 192; 67.9%). Antipsychotics were most frequently prescribed (49.5%), followed by antidepressants (39.2%) and benzodia-zepines (34.6%). Of the residents, 41.3% were prescribed PD from at least two categories and 14.1% were prescribed PD from at least three categories. A small proportion of residents only had an antipsychotic prescription (13.4%), an antidepressant prescription (8.5%) or a benzodiazepine prescription (4.6%). Some residents were prescribed two drugs within the same PD category varying from 5.0% (two antipsychotics) to 3.5% (two benzodiazepines) and 1.8% (two antidepressants) (Figure 1).

Within the category antipsychotics, according to the medication charts, atypical antipsychotics (57.1%) were prescribed more frequently than typical antipsychotics (49.3%). Of the antidepressants, SSRIs/SNRIs (63.1%) were most frequently prescribed, followed by TCAs (23.4%). Of the benzodiazepines, anxiolytics (85.7) were prescribed more frequently than hypnotics (24.5%) (Table 2).

### 3.3. Indications of Psychotropic Drug Prescriptions

Table 3 shows the indications of PDP in 184 residents according to the questionnaire. Antipsychotics were most frequently prescribed to treat a psychotic disorder (32.1%), behavioral symptoms (29.9%) or because of a combination of both (9.7%). Antidepressants were most frequently prescribed to treat a mood disorder (40.7%) or behavioral symptoms (9.3%). They were also prescribed to treat an obsessive compulsive disorder, anxiety or combinations. Benzodiazepines were most frequently prescribed to treat behavioral symptoms (26.3%), a sleep disorder (18.2%) or anxiety (14.1%). Combinations of indications were also mentioned. In 16.4% of antipsychotics, 27.8% of antidepressants and 23.2% of benzodiazepines prescriptions, the indications were unknown as filled in by the physician in the questionnaire.

### 3.4. Relationship between Behavioral Symptoms and Psychotropic Drug Prescriptions

Both antipsychotics (70.0%), antidepressants (73.0%) and benzodiazepines (75.5%) were most frequently prescribed if the symptom irritability was present, followed by agi-tation/aggression. Prescriptions of all categories of PD were also high (>50%) for disinhibition, apathy and depression. Prescriptions were given the least often for hallucinations. In a small proportion of residents, antipsychotics (1.4%) and benzodiazepines (2.0%) were prescribed while no behavioral symptom was present as measured with the NPI-Q (Table 4).

## 4. Discussion

In this study, we performed in-depth analyses of the prescriptions of PD and their indications as part of a larger cross-sectional study that examined the prevalence and severity of neuropsychiatric symptoms in residents with KS [4]. The results showed that various categories of both antipsychotics, antidepressants and benzodiazepines were prescribed to treat psychiatric disorders but also to treat behavioral symptoms or a combination of both. In particular, antipsychotics (29.9%) and benzodiazepines (26.3%) were prescribed for behavioral symptoms. Regarding the relationship with behavioral symptoms, all categories of PD were prescribed frequently (>50%) if the symptoms irritability, agitation, disinhibition, apathy or depression were present.

Studies on people with dementia showed that behavioral symptoms are associated with higher caregiver burden [17,18] and may lead to inappropriate PDP [19,20,21,22,23]. In particular, antipsychotics and benzodiazepines are associated with behavioral symptoms, and more distressing behavioral symptoms are associated with more PDP [20]. In our previous study about the prevalence and severity of neuropsychiatric symptoms in residents with KS (on the same data), we found low levels of associated caregiver distress but also extensive use of PD. The slight impact of neuropsychiatric symptoms on caregivers could be partially explained by the sedative side effect of PD [4]. This is in line with the findings in the current study, in which we found that physicians frequently prescribed PD to KS residents because of behavioral symptoms.

As far as we know, scientific evidence and practical guidelines are lacking for the treatment of behavioral symptoms in people with KS with PD. The European Medicines Agency (EMA), which is responsible for the scientific evaluation, supervision and safety monitoring of medicines in the EU [24], and the Revised Beers Criteria for Potentially Inappropriate Medications for the Elderly from the American Geriatric Society [12] do not provide recommendations to treat behavioral symptoms with PD in people with KS.

In contrast to people with KS, several guidelines to optimize the management of behavioral symptoms and the appropriate PDP in people with dementia have been developed [25,26,27]. For that purpose, the Dutch Association of Elderly Care Physicians published the ‘Multidisciplinary guideline problem behavior in dementia’ (Table 5) [28,29]. This guideline helps physicians in clinical practice through the use of a cyclic process of multidisciplinary analysis, diagnosis and treatment of behavioral symptoms with periodic evaluation. In addition, this guideline also provides recommendations for the treatment of behavioral symptoms with PD in dementia.

It is probable that Dutch physicians prescribe PD in residents with KS in accordance with the recommendations of the ‘Guideline problem behavior in people with dementia’. However, it is questionable whether this guideline is fully applicable to people with KS as this syndrome differs from dementia in socio-demographics [3], etiology and the lack of a distinct pathophysiological profile [30]. Moreover, KS has a different pattern of neurocognitive functioning and is not characterized by a progressive intellectual and cognitive decline as in dementia.

In addition, it is unclear which factors NH physicians take into consideration when prescribing PD to KS residents. Strikingly, our findings indicated that in a substantial portion of PDP, the indication was not even known by the physician (varying from 16.4% to 27.8%). From practice, it is known that professional caregivers often experience distress and a sense of incompetence in the complex care for these residents. For example, agitated behavior and the ethical challenges of how to create responsiveness in an unwilling resident could be factors that influence the sense of caregivers’ competence. This may lead to inappropriate PDP [6,31]. However, other factors, such as daytime activities and coping strategies that do not meet the needs of the resident [31,32,33] or the level of caregivers’ education, could be contributing factors to PDP.

It is the question whether PDP in KS residents can be reduced substantially. For e-xample, KS residents exhibit a relatively high prevalence of delusions as measured with the NPI-Q [4]. For instance, the resident may think that he is the director of a large company (which, in fact, is not true) or that the resident has to take care of his horses after work (which was his former occupation). This could probably be attributed to the common confabulations or a disturbance of reality monitoring [4,34]. As a consequence, people with KS might exhibit an impaired perception of reality and tend to misidentify ima-gined events as real. This might lead to delusion-like ideas. Combined with a lack of awareness, they often do not understand why they would need any help, resulting in resistance, frustration and agitation [35]. Therefore, it could be possible that PD are felt ne-cessary to treat behavioral symptoms such as suspicion and agitation. As found in our study, antipsychotics were prescribed frequently if agitation (66.4%) and delusions (33.3%) were present. Furthermore, benzodiazepines may also be useful adjuncts in the treatment of anxiety and to address alcohol craving, which is often still present.

In recent years, some non-pharmacological interventions have been developed for people with KS. One of them is ‘errorless learning’. Recent studies showed that residents with KS can become more autonomous using errorless learning principles [36,37]. In addition, this intervention was also found to have a positive effect on quality of life [38] and on reducing psychotic symptoms, affective symptoms and agitation [39]. Another intervention is the so-called ‘empathic directive approach’, which is often taught to professional caregivers working in Korsakoff wards [33]. To what extent these interventions might reduce PDP in people with KS is still unknown.

Some limitations must be addressed. Our descriptive study was mainly aimed at getting an impression whether PD are also prescribed to treat behavioral symptoms. Besides psychiatric disorders and behavioral symptoms, PD could also be prescribed for other indications such as pain, palliative sedation, epilepsy or substance use disorder. We have not investigated these indications. Furthermore, we only asked the physician whether the PD was prescribed to treat behavioral symptoms without asking deeper questions about the type of behavioral symptom. We also did not ask for the duration of the PDP. Previous studies in people with dementia showed that PDP were often inappropriately prolonged for years [19,40]. The guideline of the Dutch Association of Elderly Care suggests that physicians should regularly evaluate PDP and made an attempt to stop PDP no later than three months after starting the prescription.

## 5. Conclusions

A variety of PD are often prescribed to NH residents with KS. Besides psychiatric disorders, they are also prescribed to treat behavioral symptoms (‘off-label’). The type of behavioral symptoms and physicians’ considerations to prescribe PD to KS residents are still unknown and should be further explored. Studies on people with dementia have shown that factors related to PDP are multifactorial and include resident-, nurse- and organizational-related factors [10,41,42]. Probably these factors also influence physicians’ considerations to prescribe PD to KS residents. Furthermore, more research is needed into possible other indications for PDP and into the effectiveness and side effects of PD in people with KS. In addition, the average dosing of PD and comparison of dosing in a normal target population would be of interest and important for further studies. The insights thus obtained may, ultimately, contribute to the appropriate prescription of PD.

As long as no research has been conducted on the indications, effectiveness and side effects of PD, physicians should be cautious when prescribing PD for the treatment of behavioral symptoms in KS residents. Meanwhile, a structured, multidisciplinary approach to optimize the management of behavioral symptoms and to improve the appropriateness of PDP in people with KS is highly recommended in clinical practice. The multidisciplinary team should involve at least an elderly care physician, a psychologist and a nurse(-assistant) who are closely involved in the care of the KS resident. The presence or input of a resident’s relative is thereby strongly recommended. Non-pharmacological interventions should be seriously considered. For example, in collaboration with a psychologist’s written advice on how to approach the resident and me-thods, such as intervention sessions, peer support meetings and moral case deliberations, can support professional caregivers in dealing with behavioral symptoms.

## Figures and Tables

**Table 1:** Baseline characteristics (N = 285).

Characteristic	*n*	%
Age, mean (years)	63.1 (SD 7.9, range 41.0–84.4)	
Gender (male)	223	78.3
Marital status		
Divorced, single or widowed	245	86.0
Married or with partner	26	9.1
Unknown	14	4.9
Education		
Elementary, lower	160	56.1
Secondary	62	21.8
Higher/university	25	8.8
Unknown	38	13.3
Length of stay (years)	6.5 (SD 5.3, range 0.3–29.1)	
Somatic comorbidity		
Cardiovascular disease	109	38.3
Neurological disease	87	30.5
COPD	81	28.4
Diabetes mellitus	46	16.1
Hypertension	38	13.3
Malignancy	33	11.6
Psychiatric comorbidity		
Mood disorder	86	30.2
Schizophrenia or other psychotic disorder	48	16.8
OCD ^1^ (including hoarding)	33	11.6
Personality disorder	31	10.9
Anxiety disorder	26	9.1
Other psychiatric disorders	34	11.9
CPS, mean score (range 0–6)	2.7 (SD 1.6)	
No or mild impairment (CPS 0–1)	89	31.2
Moderate impairment (CPS 2–4)	124	43.5
Severe impairment (CPS 5–6)	68	23.9
NPI-Q, mean score (range 0–36)	8.7 (SD 5.9)	

^1^ OCD, Obsessive Compulsive Disorder.

**Table 2:** Category, type and combinations of psychotropic drug prescriptions.

Category and Type of Psychotropic Drug	Medication Chart	Questionnaire
	(N = 283)	(N = 282)
	*n* (%)	*n* (%)
Psychotropic drug (≥1)	192 (67.9)	184 (65.3)
Antipsychotic (≥1)	140 (49.5)	134 (47.5)
Typical antipsychotic	69 (49.3)	
Atypical antipsychotic	80 (57.1)	
Antidepressant (≥1)	111 (39.2)	108 (38.3)
SSRI/SNRI ^1^	70 (63.1)	
TCA ^2^	26 (23.4)	
Other	19 (17.1)	
Benzodiazepine (≥1)	98 (34.6)	99 (35.1)
Anxiolytic	84 (85.7)	
Hypnotic	24 (24.5)	
Only antipsychotic	38 (13.4)	39 (13.8)
Only antidepressant	24 (8.5)	20 (7.1)
Only benzodiazepine	13 (4.6)	10 (3.6)
Two categories of psychotropic drugs	77 (27.2)	73 (25.9)
Three categories of psychotropic drugs	40 (14.1)	42 (14.9)
Antipsychotic + antidepressant	32 (11.3)	26 (9.2)
Antipsychotic + benzodiazepine	30 (10.6)	27 (9.6)
Antidepressant + benzodiazepine	15 (5.3)	20 (7.0)
Antipsychotic + antidepressant + benzodiazepine	40 (14.1)	42 (14.9)

^1^ SSRI/SNRI, selective serotonin/norepinephrine reuptake inhibitor. ^2^ TCA, tricyclic antidepressant. Prescriptions within one drug category can be multiple.

**Table 3:** Indications of psychotropic drug prescriptions according to the questionnaire (psychotropic drug ≥ 1, N = 184).

Category	Indication	*n* (%)
Antipsychotic (n = 134)	Psychotic disorder	43 (32.1)
	Psychotic disorder and behavioral symptom	13 (9.7)
	Psychotic disorder, behavioral symptom and other indication ^1^	2 (1.5)
	Psychotic disorder and other indication ^2^	2 (1.5)
	Behavioral symptom	40 (29.9)
	Behavioral symptom and other indication ^3^	3 (2.2)
	Other indication ^4^	9 (6.7)
	Unknown	22 (16.4)
Antidepressant (n = 108)	Mood disorder	44 (40.7)
	Mood disorder and behavioral symptom	4 (3.7)
	Mood and anxiety disorder	5 (4.6)
	Mood and anxiety disorder and obsessive compulsive disorder	1 (0.9)
	Anxiety disorder	2 (1.9)
	Anxiety disorder and other indication ^5^	1 (0,9)
	Obsessive compulsive disorder	5 (4.6)
	Behavioral symptom	10 (9.3)
	Behavioral symptom and other indication ^6^	1 (1.9)
	Other indication ^7^	5 (4.6)
	Unknown	30 (27.8)
Benzodiazepine (n = 99)	Anxiety	14 (14.1)
	Anxiety and other indication ^8^	1 (1.0)
	Anxiety and behavioral symptom	3 (3.0)
	Anxiety and behavioral symptom and other indication ^9^	1 (1.0)
	Sleep disorder	18 (18.2)
	Sleep disorder and anxiety	1 (1.0)
	Sleep disorder and anxiety and other indication ^10^	1 (1.0)
	Sleep disorder and other indication ^11^	1 (1.0)
	Behavioral symptom	26 (26.3)
	Behavioral symptom and other indication ^12^	3 (3.0)
	Other indication ^13^	7 (7.1)
	Unknown	23 (23.2)

^1^ post-traumatic stress disorder (n = 1), agitation (n = 1); ^2^ psychotic disorder due to drug abuse (n = 1), paranoia (n = 1); ^3^ mood disorder (n = 1), delusions (n = 1), personality disorder (n = 1); ^4^ mood disorder (n = 2), anxiety and panic disorder (n = 1), excessive compulsive behavior (not clear OCD) (n = 1), delirium (n = 1), agitation (n = 1), anxiety (n = 1), delusions (n = 1), paranoia (n = 1); ^5^ anxiety and panic disorder (n = 1); ^6^ sadness and inactivity (n = 1); ^7^ sleep disorder (n = 2), neuropathic pain (n = 1), disinhibition (n = 1), worrying (n = 1); ^8^ anxiety (n = 1); ^9^ PTSS (n = 1); ^10^ benzodiazepine dependency (n = 1); ^11^ agitation (n = 1); ^12^ agitation (n = 2), as needed (n = 1); ^13^ epilepsy (n = 3), delusions (n = 1), alcohol withdrawal (n = 1), agitation (n = 2).

**Table 4:** Neuropsychiatric symptoms and the prescription of psychotropic drugs according to the medication chart (N = 283).

Neuropsychiatric Symptom	Antipsychotic (≥1)	Antidepressant (≥1)	Benzodiazepine (≥1)
	(N = 140)	(N = 111)	(N = 98)
	*n* (%)	*n* (%)	*n* (%)
No symptom (*n* = 10)	2 (1.4)	0	2 (2.0)
Irritability/lability	98 (70.0)	81 (73.0)	74 (75.5)
Agitation/aggression	93 (66.4)	75 (67.6)	66 (67.4)
Disinhibition	78 (55.7)	57 (51.4)	61 (62.3)
Apathy/indifference	82 (58.6)	65 (58.6)	56 (57.1)
Dysphoria/depression	71 (50.7)	62 (55.9)	61 (62.3)
Appetite/eating abnormalities	42 (30.0)	44 (39.6)	35 (35.7)
Delusions	53 (37.9)	37 (33.3)	41 (41.8)
Nighttime behavior disturbance	51 (36.4)	45 (40.5)	42 (42.9)
Anxiety	41 (29.3)	34 (30.6)	28 (28.6)
Euphoria/elation	32 (22.9)	27 (24.3)	23 (23.5)
Aberrant motor behavior	41 (29.3)	24 (21.6)	30 (30.6)
Hallucinations	17 (12.1)	15 (13.5)	11 (11.2)

**Table 5:** Recommended psychotropic drugs to treat behavioral symptoms in dementia ^1^.

Behavioral Symptom	First Choice Psychotropic Drug
Psychotic behavior	1st haloperidol ^2^, 2nd risperidone ^3^ clozapine ^3^ (Parkinson Disease) rivastigmin ^4^ (Lewy Body Disease)
Depressive behavior	1st SSRI ^5^; 2nd nortriptyline ^6^ or non-TCA
Anxious behavior	oxazepam ^7^ or lorazepam ^7^
Agitated behavior	1st haloperidol ^2^, 2nd risperidone ^3^
Apathetic behavior	rivastigmin ^4^ (Lewy Body Disease)

^1^ Multidisciplinary guideline problem behavior in dementia [28]; ^2^ typical antipsychotic; ^3^ atypical antipsychotic; ^4^ cholinesterase inhibitor; ^5^ SSRI, Selective Serotonin Reuptake Inhibitor; ^6^ TCA, tricyclic antidepressant; ^7^ short-acting benzodiazepine.

## Data Availability

Data will be available upon request to the author.
